# Association between smoking, e-cigarette use and severe COVID-19: a cohort study

**DOI:** 10.1093/ije/dyac028

**Published:** 2022-02-18

**Authors:** Min Gao, Paul Aveyard, Nicola Lindson, Jamie Hartmann-Boyce, Peter Watkinson, Duncan Young, Carol Coupland, Ashley K Clift, David Harrison, Doug Gould, Ian D Pavord, Margaret Smith, Julia Hippisley-Cox

**Affiliations:** 1 Nuffield Department of Primary Care Health Sciences, Radcliffe Observatory Quarter, University of Oxford, Oxford, UK; 2 NIHR Oxford Biomedical Research Centre, Oxford University Hospitals NHS Foundation Trust, Oxford, UK; 3 School of Public Health, Peking University Health Science Centre, Beijing, China; 4 Nuffield Department of Clinical Neurosciences, John Radcliffe Hospital, University of Oxford, Oxford, UK; 5 Faculty of Medicine & Health Sciences, University of Nottingham, Nottingham, UK; 6 Intensive Care National Audit & Research Centre (ICNARC), Napier House, London, UK; 7 Nuffield Department of Medicine, University of Oxford, Oxford, UK

**Keywords:** Smoking, E-cigarettes, COVID-19, cohort study

## Abstract

**Background:**

Smoking is a risk factor for most respiratory infections, but it may protect against SARS-CoV-2 infection. The objective was to assess whether smoking and e-cigarette use were associated with severe COVID-19.

**Methods:**

This cohort ran from 24 January 2020 until 30 April 2020 at the height of the first wave of the SARS-CoV-2 epidemic in England. It comprised 7 869 534 people representative of the population of England with smoking status, demographic factors and diseases recorded by general practitioners in the medical records, which were linked to hospital and death data. The outcomes were COVID-19-associated hospitalization, intensive care unit (ICU) admission and death. The associations between smoking and the outcomes were assessed with Cox proportional hazards models, with sequential adjustment for confounding variables and indirect causal factors (body mass index and smoking-related disease).

**Results:**

Compared with never smokers, people currently smoking were at lower risk of COVID-19 hospitalization, adjusted hazard ratios (HRs) were 0.64 (95% confidence intervals 0.60 to 0.69) for <10 cigarettes/day, 0.49 (0.41 to 0.59) for 10–19 cigarettes/day, and 0.61 (0.49 to 0.74) for ≥20 cigarettes/day. For ICU admission, the corresponding HRs were 0.31 (0.24 to 0.40), 0.15 (0.06 to 0.36), and 0.35 (0.17 to 0.74) and death were: 0.79 (0.70 to 0.89), 0.66 (0.48 to 0.90), and 0.77 (0.54 to 1.09) respectively. Former smokers were at higher risk of severe COVID-19: HRs: 1.07 (1.03 to 1.11) for hospitalization, 1.17 (1.04 to 1.31) for ICU admission, and 1.17 (1.10 to 1.24) for death. All-cause mortality was higher for current smoking than never smoking, HR 1.42 (1.36 to 1.48). Among e-cigarette users, the adjusted HR for e-cigarette use and hospitalization with COVID-19 was 1.06 (0.88 to 1.28), for ICU admission was 1.04 (0.57 to 1.89, and for death was 1.12 (0.81 to 1.55).

**Conclusions:**

Current smoking was associated with a reduced risk of severe COVID-19 but the association with e-cigarette use was unclear. All-cause mortality remained higher despite this possible reduction in death from COVID-19 during an epidemic of SARS-CoV-2. Findings support investigating possible protective mechanisms of smoking for SARS-CoV-2 infection, including the ongoing trials of nicotine to treat COVID-19.

Key Messages

- Compared with people who had never smoked, current smoking was associated with one-third lower risk of hospitalization, two-thirds lower risk of intensive care unit admission and one-fifth lower risk of death.
- People who had stopped smoking had a 10-20% higher risk of severe COVID-19.
- There was uncertain evidence on the association between e-cigarette use and severe COVID-19 because of imprecision.
- In this study, conducted at the height of English SARS-CoV-2 epidemic, current smoking was associated with higher all-cause mortality.

## Introduction

Early in the SARS-CoV-2 pandemic, smoking was identified as a risk factor for worse outcomes from COVID-19—a reasonable assumption given smoking is associated with a higher incidence of most respiratory infections.^1–3^ However, an ongoing systematic review, last updated on 13 August 2021, reported evidence from 547 studies that current smoking was associated with a reduced incidence of SARS-CoV-2.^4^ Current smoking was associated with greater risk of severe disease, with a relative risk (RR) 1.3 [[95% credibility interval (CrI) 1.01 to 1.71]. There was inconclusive evidence on whether the risks of hospitalization or death differed from those of people who have never smoked, RR 1.10 (95%CrI 0.97 to 1.24) and RR 1.13 (95%CrI 0.90 to 1.40), respectively. The same review found evidence that people who previously smoked had a similar risk of infection as did people who have never smoked but greater risk of severe COVID-19 if infected, with risk estimates suggesting that people who had stopped smoking were at greater risk than those who continued, but these were not compared directly. However, many studies of risk factors for severe COVID-19 have included only cohorts of hospitalized patients. This can induce a type of selection bias, collider bias, because several patient factors influence the likelihood of hospitalization and thereby become correlated in hospitalized patients, irretrievably biasing the strength of associations.^5^^,^^6^ We therefore need studies of largely unselected community dwelling participants with sufficient precision to determine the direction and strength of these associations.

Smoking is a risk factor for many non-communicable diseases, which are important risk factors for developing severe COVID-19.^7^ However, it is important to determine whether there is a direct causal relationship between smoking and COVID-19 outcomes. Smoking is a risk factor for disease following infection from many respiratory viruses and a natural assumption is that smoking is likely to worsen COVID-19. Key mechanisms that underlie this general susceptibility include impaired mucocilary oscillation and reduced cough reflex sensitivity leading to colonization and reproduction of pathogens. Smoke impairs the epithelium by impairing the replenishment of cells and impairing the integrity of intracellular contacts. Cell function is impaired through accumulation of mutation reducing autophagy, further impairing the respiratory barrier. Smoke decreases the activity of nitric oxide synthetase, reducing a key early defence against infection.^8–10^ Thus if smoking were to reduce the risk of SARS-CoV-2 infection or severe COVID-19, this would imply one or more constituents of tobacco smoke have specific effects against SARS-CoV-2 infection, either on the virus itself or on the immune defence structure of the lungs or more generally.^9^ Nicotine, which is widely available as a cheap pharmaceutical, is the plausible candidate component of smoke that may have this effect and its possible role is described more in the Discussion.^11^ Moreover, the SARS-CoV-2 pandemic has shown the importance of public trust in messaging, where the main weapon against the pandemic is changing population behaviour. Understanding and communicating whether smoking is a protective or a risk factor for severe COVID-19 should help maintain that trust. We therefore conducted a large community cohort study to examine the associations between smoking, nicotine use and severe COVID-19.

## Methods

### Design and participants

This was an open cohort study from 24 January 2020 until 30 April 2020 of all adults registered with 1205 general practices in England contributing to the QResearch database, representing 20% of English practices. The protocol was published.^12^ Almost everyone is registered with a general practitioner (GP), so this is a population sample. The GP database includes significant chronic diseases and information on behavioural risk factors for illness. This was linked to SARS-CoV-2 reverse transcription polymerase chain reaction (RT-PCR) test records held by Public Health England (PHE), which includes all positive cases as it is a notifiable disease. The records were linked to Hospital Episode Statistics (HES), which gives information on the diagnoses of everyone admitted to hospitals in England and the Intensive Care National Audit and Research Centre (ICNARC) Case Mix Programme database, providing data from all intensive care units (ICUs) in England.^13^ They were also linked to all death certificates in England, which include date and cause of all deaths provided by the Office for National Statistics. Outcome data were available up to 30 April 2020.

We identified a cohort of all patients aged 20 years and older who were registered with the GP practices on the start date (24 January 2020, the date of the first recorded infection in the UK). Patients entered the cohort on this date and were censored at the earliest of the following time points: date of death from unrelated causes, leaving the GP practice, occurrence of the relevant outcomes of interest, or study end date (30 April 2020). We used all the relevant patients on the pooled database to maximize power and to enhance generalizability of the results.

### Outcomes

The outcomes were:

- hospital admission for COVID-19 defined as having a positive test for SARS-CoV-2 and appearing in the HES dataset as an inpatient;
- admission to ICU with severe COVID-19 identified from ICNARC records;
- death from COVID-19 defined ICD10-codes for confirmed (U07.1) or suspected COVID (U07.2) on death certificates;
- all-cause mortality.

There was no population testing for SARS-CoV-2 taking place during this period in England, precluding analysis of the association between smoking, vaping and incidence of COVID-19 (see Supplementary material, available as Supplementary data at *IJE* online, for discussion of this).

### Exposures

GPs classified smoking status as never, former, light (1-9 cigarettes/day), moderate (10-19 cigarettes/day) or heavy smoking (≥20 cigarettes/day). We used the most recent recorded value of smoking status at study entry. We counted a person as using nicotine replacement therapy (NRT) if they had been prescribed this within 28 days of censoring. However, there were only 26 people with prescriptions of NRT in this period, meaning we could not estimate risks in this group, and they are not considered further. We classified a patient as using e-cigarettes if they had a relevant clinical code recorded in their GP record at study entry.

### Covariates

GPs in England have registers of patients and patients are not permitted to see hospital specialists, except in emergency, without GP referral. GP records are computerized and GPs record all key diseases a person has, and we used these recorded diagnoses in the analysis. Diseases are recorded at the time of diagnosis, if diagnosed by a GP, or shortly after it (e.g. from discharge summaries from hospital). We identified relevant potential confounding pathways using a directed acyclic graph (DAG). We identified that demographic factors (age, gender, socioeconomic status, ethnicity, region) and non-smoking-related disease were potential confounders. The non-smoking-related diseases were bronchiectasis, cystic fibrosis, sarcoidosis, extrinsic allergic alveolitis/hypersensitivity, other interstitial lung diseases, hypertension, type 1 diabetes, chronic liver disease, such as hepatitis, and chronic neurological conditions, such as Parkinson's disease, motor neurone disease, multiple sclerosis (MS), learning disability and cerebral palsy. Two paths reflected part of the potential causal effect of smoking on the outcome: smoking-related disease and body mass index (BMI). The smoking-related diseases were: chronic obstructive pulmonary disease (COPD), asthma, lung cancer, idiopathic pulmonary fibrosis, coronary artery disease, stroke, heart failure, atrial fibrillation, type 2 diabetes and chronic kidney disease.

In the DAG, we identified confounding paths for the association between nicotine use (vaping) and severe COVID-19 which were blocked by controlling for demographic factors (defined above) and smoking status.

### Statistical analysis

We conducted multivariable Cox proportional hazards models to estimate the unadjusted and adjusted hazard ratios (HRs) for the association between smoking status and severe COVID-19 using Stata version 16.0. We adjusted first for factors that blocked confounding associations (demographic factors and non-smoking-related diseases) and then added factors on the indirect causal paths between smoking and severe COVID-19. These were BMI (smoking reduces BMI) and smoking-related disease. This latter analysis allowed us to estimate the direct causal path between smoking and severe COVID-19 disease. In all analyses, we entered all variables as categorical variables with unrecorded data as an additional categorical variable and age as a continuous variable.

We tested whether age, gender, ethnic group and the presence of asthma or COPD modified the association between smoking and severe COVID-19. To do so, we added multiplicative interaction terms to the regression models.

We confined the analysis of the association between vaping and severe COVID-19 to people who currently or had previously smoked, as vaping is almost confined to this group. We identified that confounding would be controlled in these analyses by adjustment for demographic factors and smoking status. We re-ran the analysis including only people who were no longer smoking.

Given active smoking in the <10 cigarettes and 10-19 cigarettes a day groups was associated with reduced mortality from SARS-CoV-2 infection, the question people who smoke would face was whether to continue smoking through the pandemic or quit now. We examined all-cause mortality (including deaths due to COVID-19) using the fully adjusted model described above but combining all current smoking categories.

We conducted four post-hoc sensitivity analyses described in the Supplementary material.

## Results

There were 8 256 161 adults in the cohort, of whom 7 869 534 (95.3%) had recorded smoking status and were included in the analyses. Of these, 1 402 133 (17.8%) smoked and 1 748 966 (22.2%) had previously smoked. There were 69 047 (0.9%) recorded as using e-cigarettes, of whom 3251 (4.7%) were recorded as never smoking, 35 267 (51.1%) as formerly smoking and 30 529 (44.2%) as currently smoking. In the cohort, 14 253 (0.18%) were admitted to hospital with COVID-19, 1527 (0.019%) were admitted to ICU and there were 5817 (0.074%) deaths attributed to COVID-19. Baseline characteristics are shown in Table 1. Over half of those admitted to hospital with COVID-19 were aged over 70 years and over half of those who died were aged over 80 years. In contrast, over half of those admitted to ICU with COVID-19 were aged under 60 years.

**Table 1:** Baseline characteristics of cohort

Characteristic	Total population (*n =* 7 869 534) (% total population)	Hospitalization with COVID-19 (*n* = 14 253) (% category)	Admitted to ICU with COVID-19 (*n* = 1527) (% category)	Death from COVID- 19 (*n* = 5817) (% category)
Smoking and nicotine use				
Never smoked	4 718 435 (60.0%)	8133 (0.17%)	937 (0.02%)	3108 (0.07%)
Former smoking	1 748 966 (22.2%)	5006 (0.29%)	518 (0.03%)	2342 (0.13%)
Smoking 1 to 9 cigarettes/day	1 097 213 (13.9%)	902 (0.08%)	60 (0.01%)	295 (0.03%)
Smoking 10 to 19 cigarettes/day	208 767 (2.7%)	120 (0.06%)	5 (0.00%)	40 (0.02%)
Smoking ≥20 cigarettes/day	96 153 (1.2%)	92 (0.10%)	7 (0.01%)	32 (0.03%)
Using NRT in the month prior to censoring	26 (<0.01%)	(<0.01%)^a^	(<0.01%)^a^	(<0.01%)^a^
E-cigarette use	69 472 (0.9%)	117 (0.17%)	11 (0.02%)	41 (0.06%)
Demographics				
Mean age (SD) years	48.2 (18.6)	69.9 (17.7)	59.5 (12.5)	80.2 (12.0)
Age group				
20 to 39 years	3 325 841 (42.3%)	114 (<0.01%)	125 (<0.01%)	43 (<0.01%)
40 to 49 years	1 367 459 (17.4%)	1162 (0.08%)	198 (0.01%)	86 (0.01%)
50 to 59 years	1 354 108 (17.2%)	1892 (0.14%)	464 (0.03%)	319 (0.02%)
60 to 69 years	993 556 (12.6%)	2270 (0.23%)	457 (0.05%)	666 (0.07%)
70 to 79 years	759 838 (9.7%)	3027 (0.40%)	262 (0.03%)	1376 (0.18%)
≥ 80 years	455 359 (5.8%)	5014 (1.10%)	36 (0.01%)	3466 (0.76%)
Men	4 111 200 (52.2%)	8038 (0.20%)	1132 (0.03%)	3423 (0.08%)
Ethnicity				
White	5 359 536 (68.1%)	9193 (0.17%)	755 (0.01%)	4019 (0.07%)
Indian	227 767 (2.9%)	534 (0.23%)	81 (0.04%)	164 (0.07%)
Pakistani	148 399 (1.9%)	323 (0.22%)	52 (0.04%)	93 (0.06%)
Bangladeshi	111 077 (1.4%)	223 (0.20%)	47 (0.04%)	91 (0.08%)
Other Asian	145 010 (1.8%)	319 (0.22%)	83 (0.06%)	76 (0.05%)
Caribbean	93 339 (1.2%)	529 (0.57%)	63 (0.07%)	217 (0.23%)
Black African	198 427 (2.5%)	594 (0.30%)	129 (0.07%)	156 (0.08%)
Chinese	82 598 (1.0%)	64 (0.08%)	14 (0.02%)	25 (0.03%)
Other	306 060 (3.9%)	568 (0.19%)	113 (0.04%)	137 (0.04%)
Not recorded	1 583 948 (20.1%)	2132 (0.13%)	205 (0.01%)	978 (0.06%)
Townsend deprivation score quintile				
Quintile 1 (most affluent)	1 881 317 (23.9%)	2735 (0.15%)	245 (0.01%)	1205 (0.06%)
Quintile 2	1 821 068 (23.1%)	2810 (0.15%)	260 (0.01%)	1142 (0.06%)
Quintile 3	1 629 714 (20.7%)	2898 (0.18%)	300 (0.02%)	1292 (0.08%)
Quintile 4	1 483 531 (18.9%)	2778 (0.19%)	303 (0.02%)	1059 (0.07%)
Quintile 5 (most deprived)	1 402 402 (17.8%)	2998 (0.21%)	415 (0.03%)	1108 (0.08%)
English region				
East Midlands	220 879 (2.8%)	216 (0.10%)	15 (0.01%)	93 (0.04%)
East of England	296 231 (3.8%)	490 (0.17%)	45 (0.02%)	209 (0.07%)
London	2 059 744 (26.2%)	4963 (0.24%)	693 (0.03%)	1770 (0.09%)
North East	195 156 (2.5%)	321 (0.16%)	28 (0.01%)	109 (0.06%)
North West	1 470 043 (18.7%)	2773 (0.19%)	258 (0.02%)	1268 (0.09%)
South Central	1 104 630 (14.0%)	1720 (0.16%)	148 (0.01%)	800 (0.07%)
South East	925 796 (11.8%)	1298 (0.14%)	141 (0.02%)	577 (0.06%)
South West	900 553 (11.4%)	797 (0.09%)	68 (0.01%)	299 (0.03%)
West Midlands	777 225 (9.9%)	1 514 (0.19%)	11 (<0.01%)	654 (0.08%)
Yorkshire & Humber	305 904 (3.9%)	387 (0.13%)	35 (0.01%)	177 (0.06%)
Smoking-related morbidity				
COPD	193 520 (2.5%)	1555 (0.80%)	59 (0.03%)	811 (0.42%)
Asthma	1 090 028 (13.9%)	2266 (0.21%)	213 (0.02%)	762 (0.07%)
Lung cancer	10 792 (0.1%)	139 (1.29%)	* (<0.01%)	60 (0.56%)
Pulmonary fibrosis	7454 (0.1%)	110 (1.48%)	6 (0.08%)	62 (0.83%)
Coronary heart disease	292 839 (3.7%)	2414 (0.82%)	128 (0.04%)	1373 (0.47%)
Stroke	177 763 (2.3%)	1822 (1.02%)	51 (0.03%)	1144 (0.64%)
Atrial fibrillation	201 250 (2.6%)	1993 (0.99%)	50 (0.02%)	1100 (0.55%)
Type 2 diabetes	555 416 (7.1%)	4237 (0.76%)	480 (0.09%)	1935 (0.35%)
Chronic kidney disease	336 880 (4.3%)	3537 (1.05%)	174 (0.05%)	1962 (0.58%)
Non-smoking-related morbidity				
Bronchiectasis	41 271 (0.5%)	319 (0.77%)	18 (0.04%)	138 (0.33%)
Cystic fibrosis	2081 (<0.01%)	5 (0.24%)	(<0.01%)^a^	(<0.01%)^a^
Sarcoidosis	17 624 (0.2%)	84 (0.48%)	10 (0.06%)	32 (0.18%)
Alveolitis	2331 (0.0%)	16 (0.69%)	(<0.01%)^a^	8 (0.34%)
Interstitial lung disease	5677 (0.1%)	73 (1.29%)	(<0.01%)^a^	45 (0.79%)
Type 1 diabetes	45 947 (0.6%)	272 (0.59%)	30 (0.07%)	85 (0.18%)
Chronic liver disease	47 141 (0.6%)	286 (0.61%)	16 (0.03%)	98 (0.21%)
Chronic neurological conditions	256 538 (3.3%)	2633 (1.03%)	65 (0.03%)	222 (0.09%)
Hypertension	1 405 740 (17.9%)	7337 (0.52%)	701 (0.05%)	3570 (0.25%)
Body mass index group				
Underweight (<18.5)	220 706 (2.8%)	361 (0.16%)	5 (<0.01%)	274 (0.12%)
Healthy weight (18.5 to <25)	2 742 969 (34.9%)	3660 (0.13%)	198 (0.01%)	1869 (0.07%)
Overweight (25 to <30)	2 315 214 (29.4%)	4602 (0.20%)	491 (0.02%)	1754 (0.08%)
Obesity class I (30 to 34.9)	1 076 005 (13.7%)	2823 (0.26%)	407 (0.04%)	965 (0.09%)
Obesity class II (35 to 39.9)	406 529 (5.2%)	1276 (0.31%)	222 (0.05%)	384 (0.09%)
Obesity class III (≥40)	203 717 (2.6%)	793 (0.39%)	135 (0.07%)	234 (0.11%)
Missing BMI	1 291 021 (16.4%)	964 (0.07%)	84 (0.01%)	476 (0.04%)

NRT, nicotine replacement therapy; COPD, chronic obstructive pulmonary disease; BMI, body mass index.

a Numbers suppressed to maintain anonymity.

### Association between smoking status and severe COVID-19

After adjustment for demographic factors and non-smoking related morbidity, adults smoking fewer than 10 cigarettes/day were one-third less likely to be hospitalized, two-thirds less likely to be admitted to ICU and one-fifth less likely to die from COVID-19 than people who had never smoked (Table 2). After further adjustment for BMI and smoking-related morbidity these associations were largely unchanged. The risk estimates for people smoking 10-19 and more than 20 cigarettes/day were similar in magnitude to the group smoking fewer than 10 cigarettes daily, but estimates were less precise because 78% of people who smoked reported smoking fewer than 10 cigarettes/day (Table 1).

**Table 2:** Association between smoking status and risk of severe COVID-19

Category	Number with outcome (cumulative incidence expressed as %)	Unadjusted HR (95% CI)	HR (95% CI) adjusted for demographic factors and non-smoking-related morbidity	HR (95% CI) adjusted for demographic factors, non-smoking-related morbidity, BMI, and smoking-related morbidity
Outcome hospitalization				
Never smoked	8133 (0.17%)	1 (reference)	1 (reference)	1 (reference)
Stopped smoking	5006 (0.29%)	1.66 (1.61 to 1.72)	1.20 (1.16 to 1.25)	1.07 (1.03 to 1.11)
Smoking 1 to 9 cigarettes/day	902 (0.08%)	0.48 (0.44 to 0.51)	0.67 (0.62 to 0.72)	0.64 (0.60 to 0.69)
Smoking 10 to 19 cigarettes/day	120(0.06%)	0.33 (0.28 to 0.40)	0.50 (0.42 to 0.60)	0.49 (0.41 to 0.59)
Smoking ≥20 cigarettes/day	92 (0.10%)	0.56 (0.45 to 0.68)	0.65 (0.53 to 0.80)	0.61 (0.49 to 0.75)
Outcome ICU admission				
Never smoked	937 (0.02%)	1 (reference)	1 (reference)	1 (reference)
Stopped smoking	518 (0.03%)	1.49 (1.34 to 1.66)	1.25 (1.12 to 1.40)	1.17 (1.04 to 1.31)
Smoking 1 to 9 cigarettes/day	60 (0.01%)	0.28 (0.21 to 0.36)	0.29 (0.22 to 0.37)	0.31 (0.24 to 0.41)
Smoking 10 to 19 cigarettes/day	5 (<0.01%)	0.12 (0.05 to 0.29)	0.14 (0.06 to 0.34)	0.15 (0.06 to 0.37)
Smoking ≥20 cigarettes/day	7 (0.01%)	0.37 (0.17 to 0.77)	0.36 (0.17 to 0.75)	0.36 (0.17 to 0.76)
Outcome death				
Never smoked	3108 (0.07%)	1 (reference)	1 (reference)	1 (reference)
Stopped smoking	2342 (0.13%)	2.04 (1.93 to 2.15)	1.31 (1.24 to 1.38)	1.17 (1.10 to 1.24)
Smoking 1 to 9 cigarettes/day	295 (0.03%)	0.41 (0.36 to 0.46)	0.88 (0.78 to 1.00)	0.79 (0.70 to 0.89)
Smoking 10 to 19 cigarettes/day	40 (0.02%)	0.29 (0.21 to 0.40)	0.73 (0.53 to 1.00)	0.66 (0.48 to 0.90)
Smoking ≥20 cigarettes/day	32 (0.03%)	0.51 (0.36 to 0.72)	0.88 (0.62 to 1.25)	0.77 (0.54 to 1.10)

HR, hazard ratio; CI, confidence interval.

People who had stopped smoking had a 10-30% higher risk of COVID-19 hospitalization, ICU admission and death than people who had never smoked (Table 2). Compared with people who had stopped, people currently smoking were 40-50% less likely to be admitted to hospital for COVID-19, 70-80% less likely to be admitted to ICU and 30-40% less likely to have a COVID-19 related death.

There was evidence that the protective association between smoking and severe COVID-19 was stronger in younger people, men, ethnic minorities and people without airways disease (Supplementary material).

### Association between vaping and severe COVID-19

There were 69 472 people who used e-cigarettes, of whom 65 796 (95%) had previously or currently smoked. There was no evidence that e-cigarette use was associated with a difference in risk of severe COVID-19, but the estimates were imprecise, encompassing from modest protection to substantial increased risk. We confined the analysis to the 35 267 people not currently smoking and the same pattern was observed (Table 3).

**Table 3:** Association between e-cigarette use and severe COVID-19 outcomes among people who currently or previously smoked

	People currently or previously smoking (*n* = 3 151 099)			People who had previously smoked (*n* = 1 748 966)		
	Number with outcome (cumulative incidence expressed as %)	Unadjusted HR (95% CI)	HR (95% CI) adjusted for demographic factors and smoking	Number with outcome	Unadjusted HR (95% CI)	HR (95% CI) adjusted for demographic factors and smoking
Outcome hospitalization						
Not using e-cigarettes	6011 (0.19%)	1 (reference)	1 (reference)	4934(0.29%)	1 (reference)	1 (reference)
Using e-cigarettes	109 (0.17%)	0.85 (0.70 to 1.03)	1.06 (0.88 to 1.28)	72(0.20%)	0.71 (0.56 to 0.89)	1.02 (0.81 to 1.29)
Outcome ICU admission						
Not using e-cigarettes	579 (0.02%)	1 (reference)	1 (reference)	507(0.03%)	1 (reference)	1 (reference)
Using e-cigarettes	11 (0.02%)	0.89 (0.49 to 1.62)	1.04 (0.57 to 1.89)	11(0.03%)	1.05 (0.58 to 1.91)	1.20 (0.66 to 2.20)
Outcome death						
Not using e-cigarettes	2671 (0.09%)	1 (reference)	1 (reference)	2318(0.14%)	1 (reference)	1 (reference)
Using e-cigarettes	38 (0.06%)	0.67 (0.48 to 0.92)	1.12 (0.81 to 1.55)	24(0.07%)	0.50 (0.34 to 0.75)	1.03 (0.69 to 1.54)

ICU, intensive care unit.

### All-cause mortality

There were 27 739 deaths in total, including 5817 from COVID-19. In people currently smoking, the corresponding figures were 3053 and 367, and in people who had stopped smoking were 10 381 and 2342. The HR for current smoking was 1.42 (1.36 to 1.48) and for previous smoking was 1.11 (1.08 to 1.14) compared with never smoking. There was heterogeneity by age (*P* <0.0001), however, with all age groups showing increased risk of death associated with smoking except for those over 80 years, where the HR was 0.89 (0.83 to 0.95).

### Post-hoc sensitivity analyses

See the Supplementary material for full methods, results and discussion. Excluding people who lived in care homes from the analysis of death gave similar results. Analyses using smoking status from 5 years previously showed that current smoking was associated with a reduced risk of hospitalization and ICU admission. However, current smoking from 5 years previously was associated with an increased risk of death from COVID-19. Modelling age as a non-linear term using restricted cubic splines produced results that were very similar to those above. We stratified by (rather than adjusted for) the presence of smoking-related disease and produced estimates for people with and without smoking-related disease, showing very similar outcomes for each group as for both groups combined.

## Discussion

### Summary

In this large community cohort, current smoking was associated with a lower risk of severe COVID-19 outcomes and this was not explained by associated demographic or non-smoking-related or smoking-related morbidity. Compared with people who had never smoked, the risk of hospitalization was reduced by one-third, the risk of admission to ICU by two-thirds and the risk of death by one-fifth. In the same analyses, having stopped smoking was associated with a risk of death that was 10–20% higher than for people who had never smoked. There was effect modification, with the risk reductions seen with current smoking being greater in men than women, in younger people than older and in people without airways disease compared with those with it. However, during the height of the English SARS-CoV-2 epidemic, all-cause mortality was higher in people who smoked than in those who had stopped despite the risk reduction in COVID-19 deaths observed.

### Strengths and limitations

The strengths of this study include the large representative community cohort giving precise estimates of association which are likely to be generalizable to the whole UK population. In addition, smoking status was measured prior to developing COVID-19 outcomes, unlike most studies in the an ongoing systematic review^4^ which assessed smoking status on admission to hospital. People who are becoming breathless with COVID-19 are likely to at least suspend smoking and may declare that they have stopped smoking on admission, thus giving rise to an apparent lower risk from smoking and higher risk from having stopped smoking. That said, the prevalence of current smoking recorded in this study of medical records (17.8%) is higher than the current English smoking prevalence (14.1%).^14^ This reflects that GPs recorded smoking status a median of 2 years prior to study end, when the prevalence was higher. Thus, some people who were recorded as currently smoking would subsequently have stopped smoking, and fewer people recorded as having stopped are likely to have relapsed, resulting in a reclassification bias. This is likely to bias estimates towards the null.

A key concern with observational epidemiological studies is that associations arise because of unmeasured or residual confounding. Here, the E-value for the associations for people smoking 1–9 cigarettes/day are 2.5 for hospitalization, 5.9 for ICU admission and 1.9 for death, with lower confidence interval E-values 2.3, 4.3 and 1.5, respectively..^15^^,^^16^ This means that an unmeasured confounder would need to be 2.5 times as common among people who smoked 1–9 cigarettes/day compared with never smokers, and to reduce risk of hospitalization 2.5-fold to explain the association between smoking and hospitalization. (A full range of E-values are given in the Supplementary material, compared with those calculated from a study of the harms of smoking. This showed E-values for well-established smoking-related illnesses were of similar magnitude.) People who smoked were on average 11 years younger than people who used to smoke and 2 years younger than people who never smoked, and younger people are at substantially less risk of severe COVID-19. However, we adjusted for age as a continuous variable in our analyses. Alternatively, omitted comorbidities which raise the risk of severe COVID-19 would need to be 2.5-fold more common among people who have never smoked than in people currently smoking and to confer a 2.5-fold risk of hospitalization. However, smoking protects against very few diseases and the prevalence of these in the population makes residual confounding by such conditions unlikely. Moreover, an unmeasured confounder would need to be distributed such that it increased the apparent risk in people who have stopped smoking but decreased the apparent risk in people who currently smoke. Differences by patients in their preferences or doctors in their referral behaviour for hospital or ICU care could be related to smoking status, but death as an outcome would not, so that the latter analysis should be unbiased by selection factors.

A weakness of these data is that e-cigarette use was probably underestimated by GP records, with 0.8% recorded as using e-cigarettes, when the national prevalence is 5%.^17^ Although most users of e-cigarettes are likely to be classified as not using them, they are greatly outnumbered by people who are genuinely not using e-cigarettes, and this is unlikely to distort the association meaningfully. The more serious bias would arise if people recorded as using e-cigarettes were no longer using them. Vaping status was recorded a median of 23 (interquartile range 38 to 10) months prior to study start. One cohort suggested that over half of initial users have ceased e-cigarette use after 6 months.^18^ If e-cigarette use were protective, then including former users in this group would dilute the effect estimate.

In this study, we were unable to assess the association between smoking and incidence of COVID-19 because population testing was not conducted during the study period. One study suggested that only around 3% of older people with COVID-19 symptoms were admitted to hospital.^19^ Thus, we are unable to assess whether smoking may protect against infection with SARS-CoV-2 or alter the course of disease to reduce its severity in people with infection. That said, it is the incidence of severe COVID-19 that is putting enormous strain on hospitals in countries with active SARS-CoV-2 epidemics, not the incidence of much more common but milder COVID-19. Arguably therefore, severe disease is the most relevant outcome to assess. Hospital testing was universal during this period, which means that differences between people who smoke and those who do not in testing are unlikely to greatly affect our findings here. However, around one-fifth of deaths occurred in people who were never admitted to hospital, often very frail with multimorbidity and living in care homes. As such, the diagnosis of death from COVID-19 was clinical and not microbiological, but misattribution of cause of death is unlikely to be differentially related to smoking status.

We found that the association between smoking and reduced risk of severe COVID was greater in younger people. This may reflect that older people had already developed early-stage and undiagnosed smoking-related morbidity, which reduced the apparent protection. Likewise, the association was slightly stronger in men than women. These exploratory findings need confirmation.’

### Results in context with other literature

The systematic review of the association between smoking and COVID-19 reported that smoking prevalence in people with COVID-19 was below the national smoking prevalence in most studies, and former smoking prevalence was similar to the national prevalence.^4^ The meta-analysis reported in the Introduction suggested that current smoking was associated with a lower risk of infection than never smoking but uncertainty over more severe disease.^4^ However, all these data were not adjusted for confounding, which is a particular concern because the age profiles of current, former and never smokers are likely to differ. Our data therefore extend these findings considerably, by providing a community cohort with prospective registration of smoking status and a range of markers of severe COVID-19. A population study similar to ours showed a reduced risk of COVID-19 death associated with current smoking in an analysis adjusted for smoking-related morbidity, with an HR of 0.89 (0.82 to 0.97).^7^ Taken together, the data suggest that smoking is associated with a reduced risk of infection with SARS-CoV-2 and severe COVID-19, but it remains unclear whether nicotine use without smoking is associated with a similar reduction in risk. However, a recent study combined observational analysis and Mendelian randomiaztion data using the UK Biobank cohort and reported that current smoking was associated with elevated risk of severe COVID-19.^20^ Only around 3% of the cohort smoked as this was a population implicitly selected by interest in health, which clouds the observational data. Such concerns should not apply to the Mendelian randomiation data, however, and the conflicting results are hard to reconcile.

It would be possible to explain the results in our study and the systematic review through behaviour. People who smoke were warned about increased risk from smoking and may have been more rigorously social distancing. However, a cross-sectional study in England reported that people who smoked had an odds ratio of 0.70 (0.62 to 0.78) for adhering to Government guidelines, with an odds ratio of 4.9 (4.4 to 5.4) for ‘living life as normal’.^21^

### Implications

Our data and at least some of the accumulated evidence may suggest that cigarette smoke contains one or more chemicals that reduce risk, and nicotine is a plausible candidate with several possible mechanisms. One study reported that nicotine downregulates the ACE2 receptor, to which SARS-CoV-2 attaches,^22^ but other studies have found the opposite.^23^ Nicotine may also be anti-inflammatory. Nicotinic alpha-7 agonists reduce the severity of pancreatitis and peritonitis in animal models,^24^^,^^25^ with infusions of nicotine causing decreased immune cell influx and reduced pro-inflammatory cytokine release, which may be relevant in COVID-19. Smoking alters the distribution of T-helper cells, particularly shifting the balance between Th1, Th2 and Th17 cells, thereby influencing cytokine release, a crucial part of severe COVID-19 disease.^26^ Alpha-4 and alpha-7 nicotinic receptors play important roles in regulating B cell lines.^26^ Nicotine administration reduces the proliferation of cytotoxic T lymphocytes.^27^ Stimulation of alpha-7 nicotinic acetylcholine receptors activates murine macrophages inhibiting the transcription of pro-inflammatory cytokines, which may be relevant in COVID-19.^28^ Furthermore, activated α7 neuronal nicotinic acetylcholine receptors (nAChR) suppressed the phosphorylation of Ikappa B kinase (IκB), downregulating nuclear translocation of nuclear factor kappa-light-chain-enhancer of activated B cells (NFκB).^29^^,^^30^ NFκB is activated in response to interleukin-1 (IL-1) and tumour necrosis factor alpha (TNF-alpha) release and in turn enables expression of cytokines, chemokines and other adhesion molecules.^31^ These mechanisms may partially explain why nicotine can induce remission in ulcerative colitis.^32^ In our study there was no evidence, however, that people consuming nicotine without smoking were at reduced risk, but further preclinical and clinical studies should examine this.

From the start of the SARS-CoV-2 pandemic, people who smoke have been warned of their excess risk from COVID-19. However, although there are contrary studies, the body of studies does not generally show there to be any substantial risk. In England, record numbers of people have stopped smoking during the SARS-COV-2 epidemic,^33^ which could be as a result of heightened health concerns. Our data join many other studies in showing the risks of smoking and the benefits of quitting on mortality^34^ where, even at the height of the English SARS-CoV-2 epidemic, all-cause mortality remained higher among people currently smoking than those who had stopped, despite a lower death rate from COVID-19. However uncomfortable, public messaging on smoking should reflect the state of scientific evidence: honesty over which appears to be paramount in maintaining public trust.^35^ Whereas there are no direct data suggesting that nicotine is the compound in cigarette smoke that may protect against SARS-CoV-2, these results and previous experience with nicotine in inflammatory bowel disease support testing nicotine as a treatment for COVID-19.

## Conclusions

Current smoking was associated with a reduced risk of severe COVID-19 but the association with e-cigarette use was unclear. All-cause mortality remained higher despite this possible reduction in death from COVID-19 during an epidemic of SARS-CoV-2. Findings support investigating possible protective mechanisms of smoking for SARS-CoV-2 infection, including the ongoing trials of nicotine to treat COVID-19.

## Ethics approval

The study was approved by the QResearch Science committee, which has delegated power from Derby Research Ethics Committee (Ref: 18/EM/0400) to approve studies using QResearch.

## Data availability

The data belong to the NHS and cannot be shared.

## Supplementary data

Supplementary data are available at *IJE* online.

## Author contributions

J.H.C. and P.W. conceived the record linkages and developed the platforms for the overall study. N.L., J.H.B., P.A. and M.S. conceived this analysis. All authors discussed the protocol and edited it. M.G. and P.A. developed the analysis plan and M.G. analysed the data. P.A. wrote the manuscript and all other authors contributed to it. M.G. and P.A. are guarantors.

## Funding

These linkages were paid for by the NIHR Oxford Biomedical Research Centre and Wellcome Trust, which had no role in the design, analysis or decision to submit for publication.

## Supplementary Material

dyac028_Supplementary_Data
