# Supplementary material for: Association between smoking, e-cigarette use and severe COVID-19: a cohort study
Source: Int J Epidemiol. 2022 Feb 18;51(4):1062–72. doi: 10.1093/ije/dyac028 (PMC8903448; doi:10.1093/ije/dyac028)
Supplement: dyac028_Supplementary_Data [file dyac028_supplementary_data.docx]

**Supplementary Material**

**Contents**

Patient and public involvement

Modification of the association between smoking and severe COVID-19 by demographic factors and smoking-related respiratory disease

Figure S1 Effect modification of the association between smoking and severe COVID-19 by age for hospitalization, ICU admission, and death (panels left to right)

Figure S2 Effect modification of the association between smoking and severe COVID-19 by gender, ethnicity, and the presence of COPD or asthma for hospitalization, ICU admission*, and death*

Post-hoc sensitivity analyses methods

Figure S3 Restricted cubic splines for association between age and COVID-19 Hospital Admission, admission to ICU, and death

Table S1 Association between smoking status and risk of severe COVID-19 using smoking status recorded at least 5 years prior to study end (N=6,601,690)

Table S2 Association between smoking status and risk of severe COVID-19 with modelling age as non-linear with restricted cubic splines

Table S3 Association between e-cigarette use and severe COVID-19 outcomes among people who currently or previously smoked with modelling age as non-linear

Table S4 Association between smoking status and risk of severe COVID-19 in people without smoking-related disease (N=5,751,061)

Table S5 Association between smoking status and risk of severe COVID-19 in people with smoking-related disease (N=2,118,473)

Post hoc sensitivity analysis discussion

Table S6 E-values corresponding to Table 2

Table S7 Established smoking-related disease and their E-values from the paper

**Patient and public involvement**

We discussed the rationale for the study with members of the public who form part of NIHR Oxford Biomedical Research Centre’s public and patient panel. They agreed that the study rationale was appropriate and provided editorial advice on the lay summary of the protocol. We then discussed the results, and, in particular about presenting results to the public in light of the findings. Members of the public advised on the framing of risk and were keen to ensure that findings on all-cause mortality were presented alongside reductions in the risk of severe COVID-19.

***Modification of the association between smoking and severe COVID-19 by demographic factors and smoking-related respiratory disease***

Given there was no strong evidence of a dose-response relation and that 78% of people who smoked consumed fewer than 10 cigarettes daily, we combined all current smoking groups for these analyses for clarity and to maintain power. There was strong statistical evidence of effect modification between smoking and demographic characteristics, although the difference in strength of association was modest. The reduction in risk seen in people who smoked was stronger in people under 60 years (Supplementary material Figure 1), while previous smoking was associated with a less elevated risk among younger compared with older people. Gender also modified the association: the reduction in risk of severe COVID-19 outcomes associated with smoking was greater in men than women (Supplementary material Figure 2). There was also strong statistical evidence of effect modification by ethnicity, though the pattern was less consistent (Supplementary material Figure 4). The most prominent finding was that the risk reduction associated with smoking was greater for people of Asian ethnicity for all outcomes.

There was also some evidence that having COPD or asthma modified the association between smoking and severe COVID-19 (Supplementary material Figure 4). Having COPD or asthma reduced the strength of association between smoking and COVID-19 hospitalization, but there was no evidence of effect modification for ICU admission or death. Having stopped smoking was associated with slightly greater risk of severe COVID-19 in people with COPD or asthma than without (Supplementary material Figure 4).

**Figure S1 Effect modification of the association between smoking and severe COVID-19 by age for hospitalization, ICU admission, and death (panels left to right)**


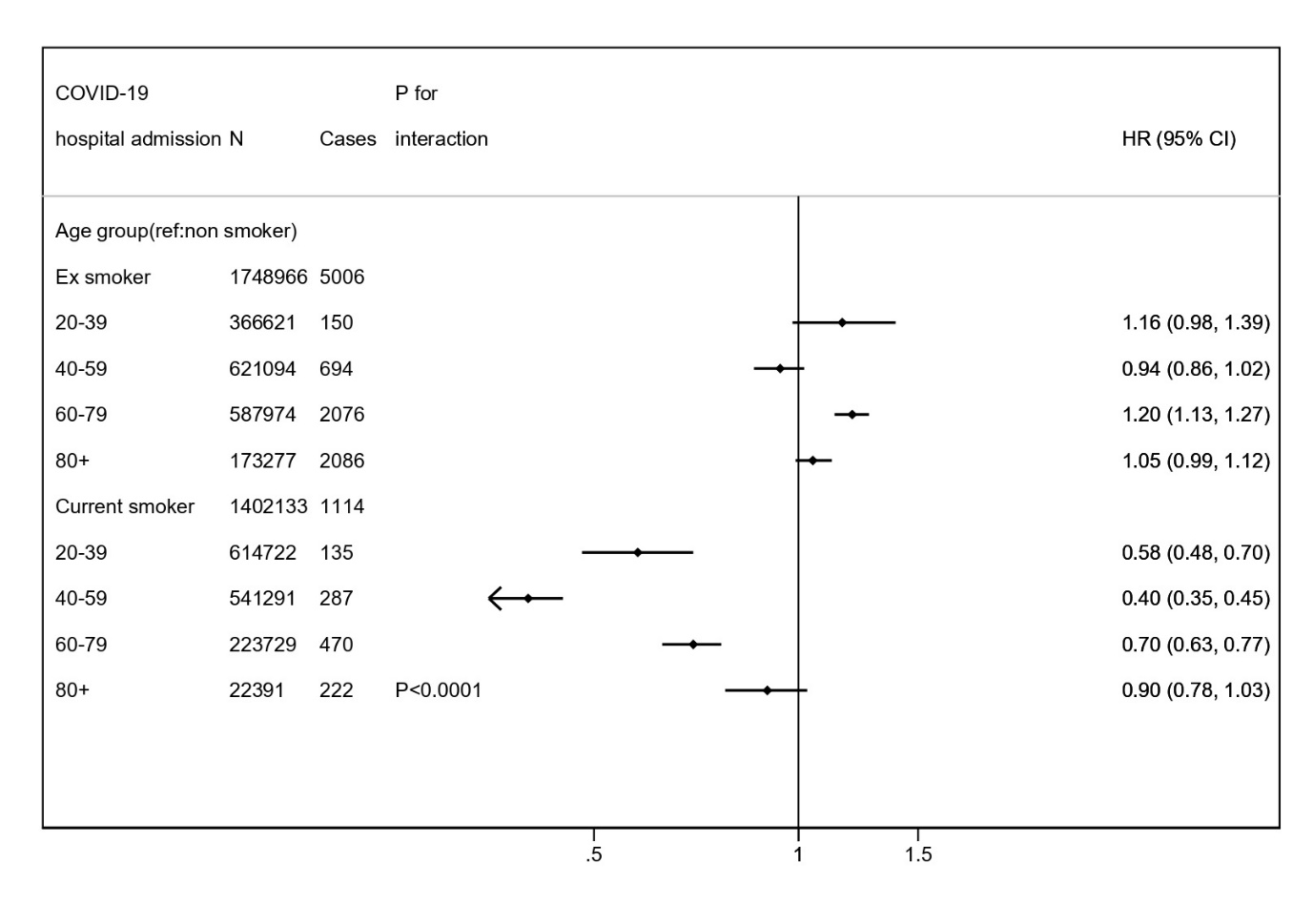


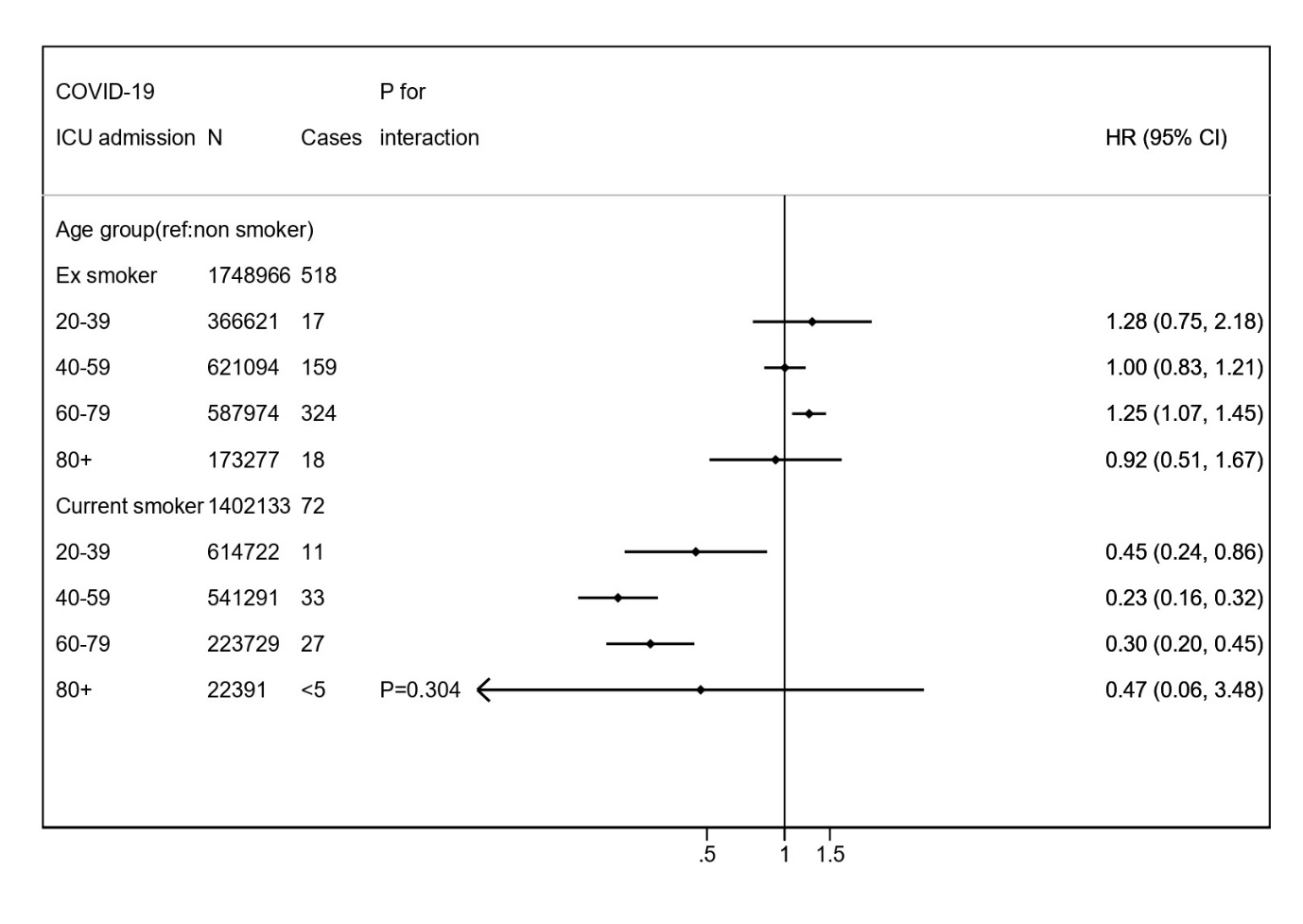


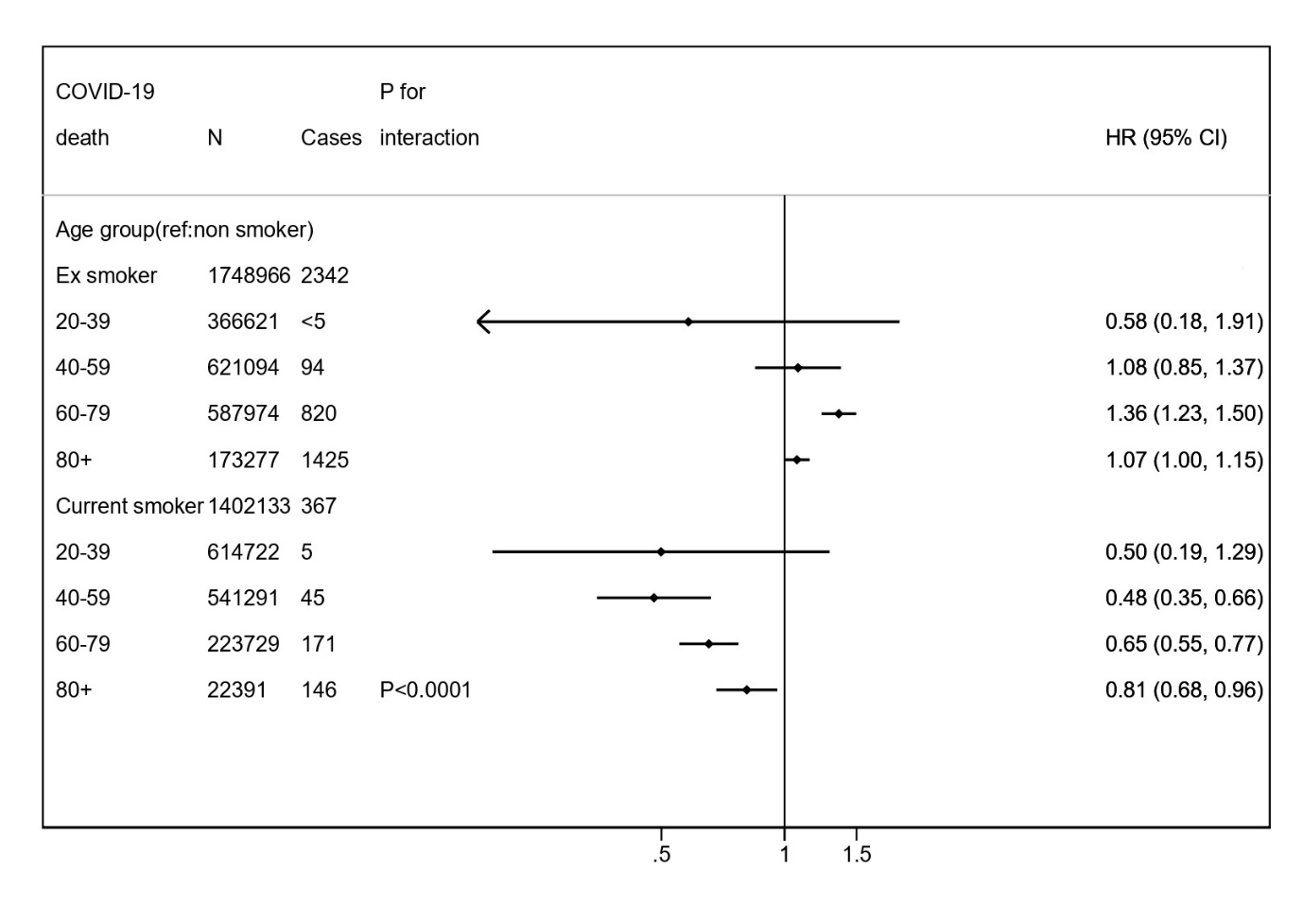


These models were adjusted for demographic factors, non-smoking-related morbidity, BMI, and smoking-related morbidity

**Figure S2 Effect modification of the association between smoking and severe COVID-19 by gender, ethnicity, and the presence of COPD or asthma for hospitalization, ICU admission*, and death***

**
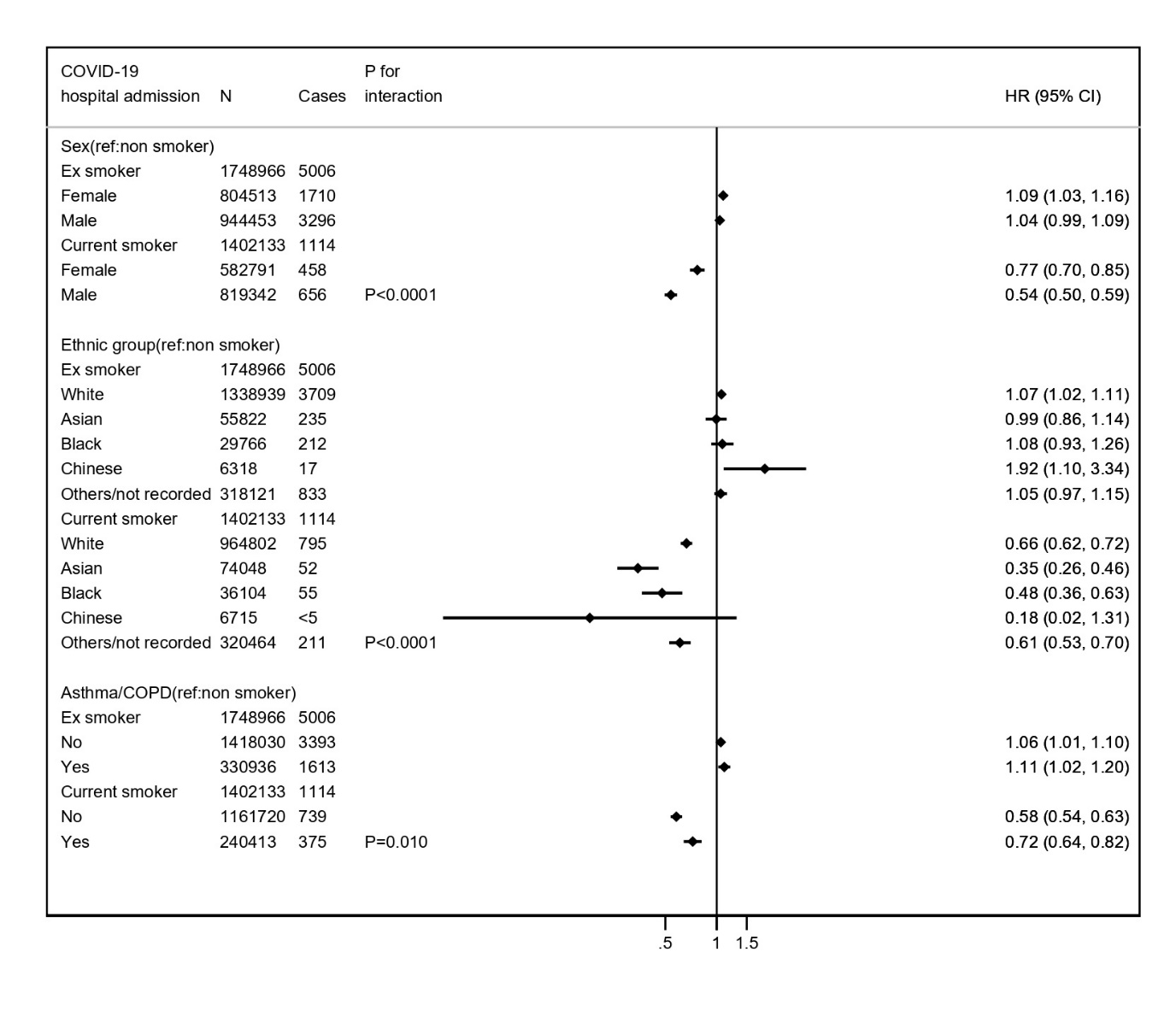
**

**
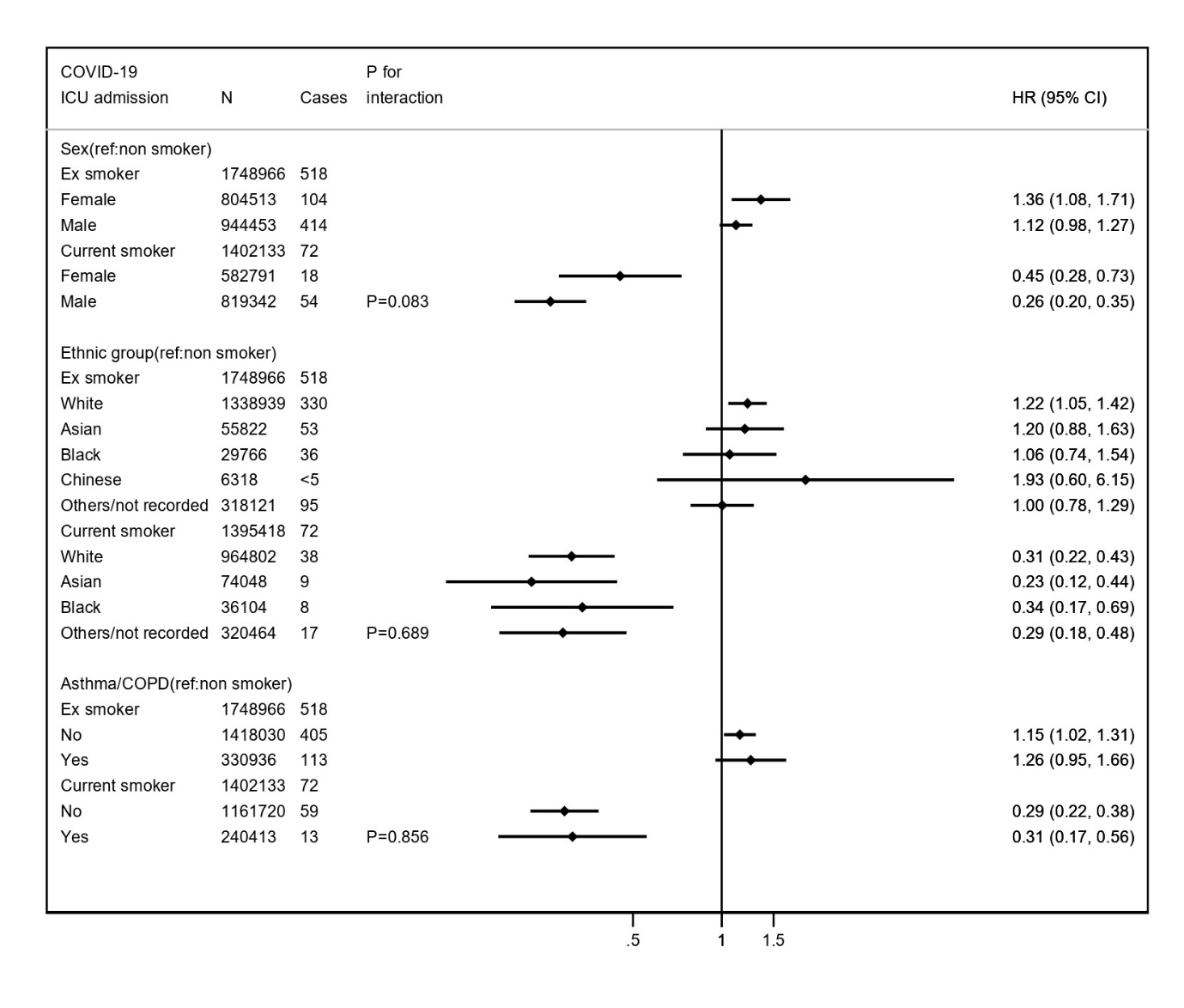
**

**
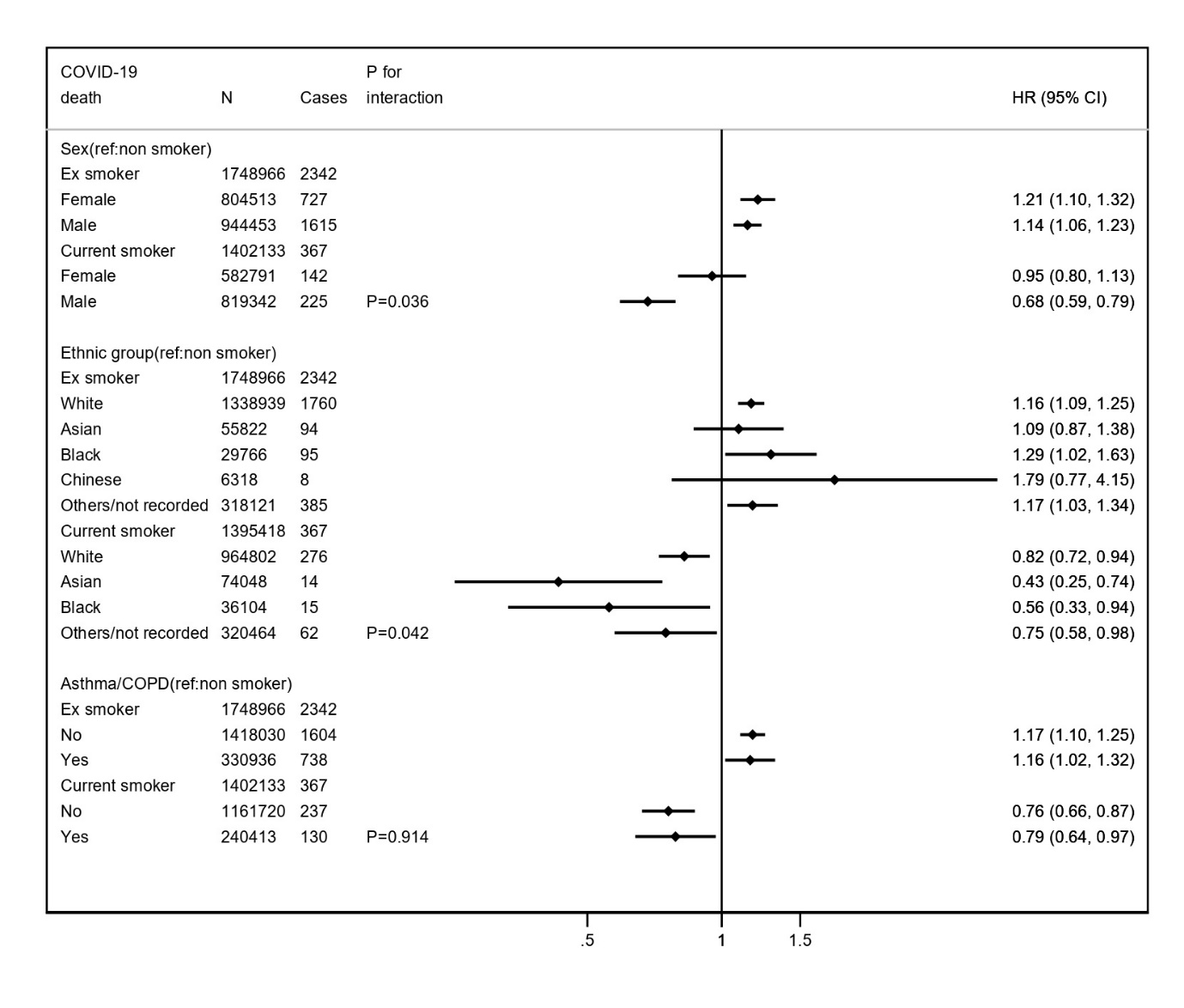
**

*Chinese people excluded because there were no cases

These models were adjusted for demographic factors, non-smoking-related morbidity, BMI, and smoking-related morbidity

***Post-hoc sensitivity analyses methods***

Colleagues and referees raised three concerns with these counter-intuitive results. First, serious outcomes from SARS-CoV-2 infection were particularly common in people living in care homes. Such people are commonly frail and simply adjusting for prevalent disease will not adequately capture the risk this poses. Moreover, smoking among care home residents is likely to be rare because of a combination of frailty and laws against smoking inside in England. We therefore excluded such residents in this sensitivity analysis. There were 49,184 (0.6%) people resident in care homes. This group gave rise to 8% of all hospital admissions, <1% of all ICU admissions, but 25% of all COVID-19 deaths, so we confined this analysis to death as the outcome. The fully adjusted analyses gave HRs for current smoking and COVID-19 deaths of 0.88 (0.77 to 1.00) adjusted for demographic factors and non-smoking related disease and 0.81 (0.71 to 0.91) additionally adjusted for smoking-related disease. For all-cause mortality, the HRs were 1.92 (1.84 to 2.01) adjusted for demographics and non-smoking-related disease and 1.57 (1.50 to 1.64) additionally adjusted for smoking-related disease.

A second concern is reverse causation, whereby people who smoke and are showing symptoms of serious illness stop smoking due to heightened health concerns. Such people are likely to visit their GP where their new smoking status is recorded, while their underlying but undiagnosed disease places them at high risk of serious COVID-19. We examined this in two ways. First, we assessed the date of recording the latest smoking status used in our analyses. Smoking status was recorded a median (interquartile range) of 19 months (7 to 50 months) prior to study start. Second, we reanalyzed the data using smoking status recorded at least 5 years prior to study end, with the exposure being smoking status as a three-category variable. In doing so, 1,654,471 (20.0%) had a missing smoking status but among those with recorded smoking status, 19.1% were smoking, 21.1% had previously smoked, and 59.9% never smoked. Smoking was recorded a median (interquartile range) of 75 (66 to 96) months before study start. Table S1 shows that the HRs for all outcomes were moved towards the null for people classified as currently smoking for hospitalization and ICU admission, while current smoking was positively associated with COVID-19 death.

A third concern relates to residual confounding by age. Age is the strongest risk factor for severe COVID-19 and we specified a linear (log linear) term for age, which may not fit the data well. We therefore fitted restricted cubic splines with 5 knots. Five knots were specified to provide enough flexibility to model the risk with age, whilst also not being too many knots so that the model is over-sensitive to the smallest fluctuations. We then reran the regression models using the splines to account for confounding by age. The splines for the association between age and outcome are shown below (Figure S3).

The editors of the International Journal of Epidemiology asked for the analysis stratified by the presence or absence of smoking-related disease rather than adjusting for it.

**Figure S3 Association between age and risk of hospital admission, admission to ICU, and death**


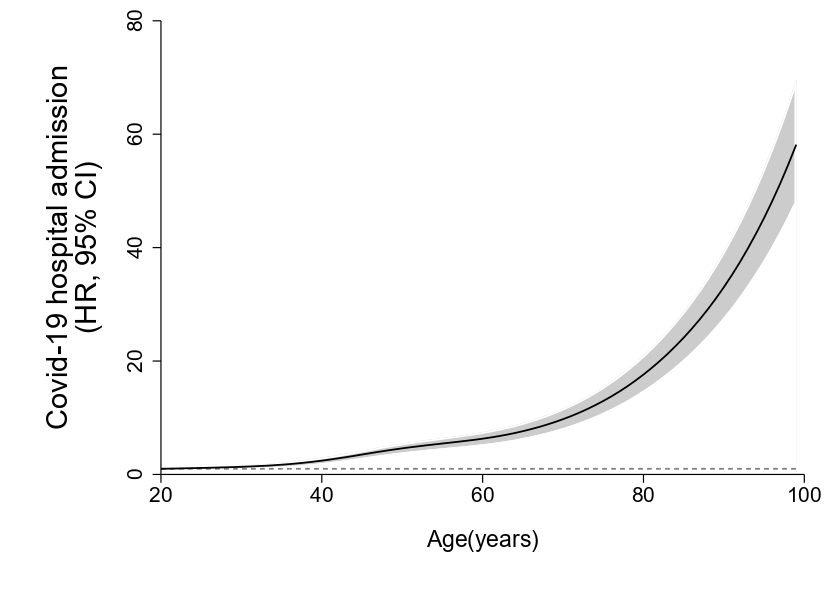


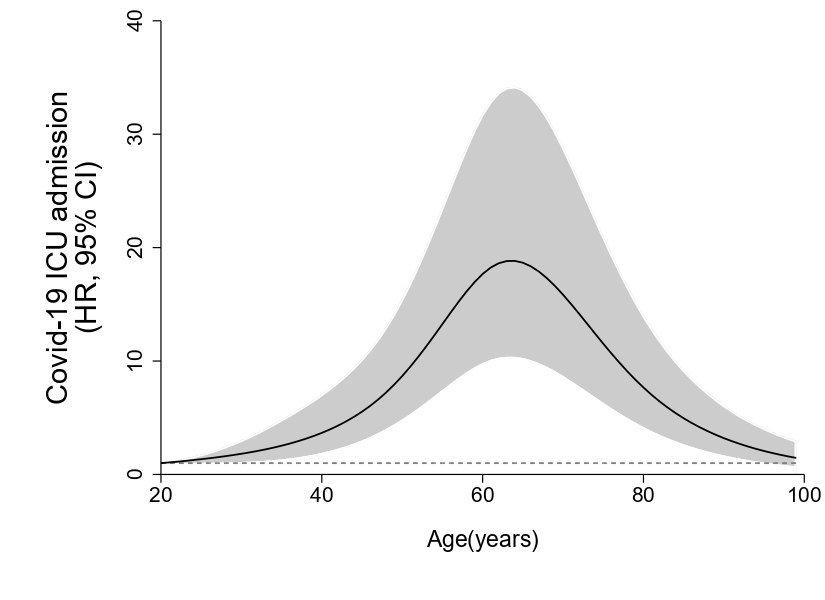


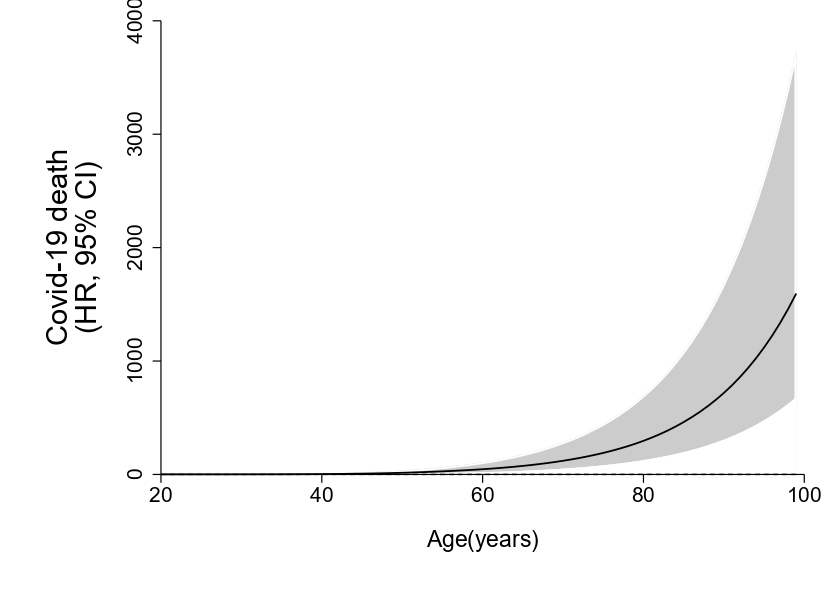


**Table S1 Association between smoking status and risk of severe COVID-19 using smoking status recorded at least 5 years prior to study end (N=6,601,690)**

| Category | Number with outcome | Unadjusted HR (95% CI) | HR (95% CI) adjusted for demographic factors and non-smoking related morbidity | HR (95% CI) adjusted for demographic factors, non-smoking-related morbidity, BMI, and smoking-related morbidity |
| --- | --- | --- | --- | --- |
| Outcome hospitalization |  |  |  |  |
| Never smoked | 7,044(0.18%) | 1 (reference) | 1 (reference) | 1 (reference) |
| Stopped smoking | 4,308(0.31%) | 1.74 (1.67 to 1.81) | 1.23 (1.18 to 1.28) | 1.10 (1.06 to 1.15) |
| Currently smoking | 1,471(0.12%) | 0.66 (0.62 to 0.69) | 0.87 (0.82 to 0.92) | 0.83 (0.78 to 0.88) |
| Outcome ICU admission |  |  |  |  |
| Never smoked | 814(0.02%) | 1 (reference) | 1 (reference) | 1 (reference) |
| Stopped smoking | 452(0.03%) | 1.58 (1.41 to 1.77) | 1.34 (1.18 to 1.51) | 1.24 (1.10 to 1.40) |
| Currently smoking | 123(0.01%) | 0.47 (0.39 to 0.57) | 0.49 (0.41 to 0.60) | 0.53 (0.44 to 0.64) |
| Outcome death |  |  |  |  |
| Never smoked | 2,524(0.06%) | 1 (reference) | 1 (reference) | 1 (reference) |
| Stopped smoking | 2,008(0.14%) | 2.26 (2.13 to 2.40) | 1.40 (1.32 to 1.49) | 1.26 (1.19 to 1.35) |
| Currently smoking | 600(0.05%) | 0.75 (0.68 to 0.82) | 1.43 (1.30 to 1.57) | 1.30 (1.18 to 1.43) |

**Table S2 Association between smoking status and risk of severe COVID-19 with modelling age as non-linear with restricted cubic splines**

| Category | Number with outcome | Unadjusted HR (95% CI) | HR (95% CI) adjusted for demographic factors and non-smoking related morbidity | HR (95% CI) adjusted for demographic factors, non-smoking-related morbidity, BMI, and smoking-related morbidity |
| --- | --- | --- | --- | --- |
| Outcome hospitalization |  |  |  |  |
| Never smoked | 8133 | 1 (reference) | 1 (reference) | 1 (reference) |
| Stopped smoking | 5006 | 1.66 (1.6 to 1.72) | 1.21 (1.17 to 1.26) | 1.07 (1.03 to 1.12) |
| Smoking 1-9 cigarettes/day | 902 | 0.48 (0.45 to 0.51) | 0.67 (0.63 to 0.72) | 0.64 (0.60 to 0.69) |
| Smoking 10-19 cigarettes/day | 120 | 0.33 (0.28 to 0.40) | 0.51 (0.42 to 0.61) | 0.49 (0.41 to 0.59) |
| Smoking ≥20 cigarettes/day | 92 | 0.55 (0.45 to 0.68) | 0.66 (0.53 to 0.81) | 0.61 (0.50 to 0.75) |
| Outcome ICU admission |  |  |  |  |
| Never smoked | 937 | 1 (reference) | 1 (reference) | 1 (reference) |
| Stopped smoking | 518 | 1.49 (1.34 to 1.66) | 1.21 (1.08 to 1.35) | 1.15 (1.02 to 1.28) |
| Smoking 1-9 cigarettes/day | 60 | 0.28 (0.21 to 0.36) | 0.27 (0.20 to 0.35) | 0.30 (0.23 to 0.38) |
| Smoking 10-19 cigarettes/day | 5 | 0.12 (0.05 to 0.29) | 0.12 (0.05 to 0.30) | 0.14 (0.06 to 0.34) |
| Smoking ≥20 cigarettes/day | 7 | 0.37 (0.17 to 0.77) | 0.29 (0.14 to 0.60) | 0.31 (0.15 to 0.65) |
| Outcome death |  |  |  |  |
| Never smoked | 3108 | 1 (reference) | 1 (reference) | 1 (reference) |
| Stopped smoking | 2342 | 2.04 (1.93 to 2.15) | 1.30 (1.23 to 1.37) | 1.16 (1.10 to 1.23) |
| Smoking 1-9 cigarettes/day | 295 | 0.41 (0.36 to 0.46) | 0.88 (0.78 to 0.99) | 0.78 (0.69 to 0.89) |
| Smoking 10-19 cigarettes/day | 40 | 0.29 (0.21 to 0.40) | 0.73 (0.53 to 1.00) | 0.65 (0.48 to 0.90) |
| Smoking ≥20 cigarettes/day | 32 | 0.51 (0.36 to 0.72) | 0.87 (0.62 to 1.23) | 0.75 (0.53 to 1.07) |

**Table S3 Association between e-cigarette use and severe COVID-19 outcomes among people who currently or previously smoked with modelling age as non-linear**

|  | People currently or previously smoking (n=3,151,099) | | | People who had previously smoked (n=1,748,966) | | |
| --- | --- | --- | --- | --- | --- | --- |
|  | Number with outcome | Unadjusted HR (95% CI) | HR (95% CI) adjusted for demographic factors and smoking | Number with outcome | Unadjusted HR (95% CI) | HR (95% CI) adjusted for demographic factors and smoking |
| Outcome hospitalization |  |  |  |  |  |  |
| Not using e-cigarettes | 6011 | 1 (reference) | 1 (reference) | 4934 | 1 (reference) | 1 (reference) |
| Using e-cigarettes | 109 | 0.85 (0.70 to 1.03) | 1.08 (0.90 to 1.32) | 72 | 0.71 (0.56 to 0.89) | 1.03 (0.82 to 1.30) |
| Outcome ICU admission |  |  |  |  |  |  |
| Not using e-cigarettes | 579 | 1 (reference) | 1 (reference) | 507 | 1 (reference) | 1 (reference) |
| Using e-cigarettes | 11 | 0.89 (0.49 to 1.62) | 1.04 (0.46 to 1.53) | 11 | 1.05 (0.58 to 1.92) | 1.00 (0.55 to 1.81) |
| Outcome death |  |  |  |  |  |  |
| Not using e-cigarettes | 2671 | 1 (reference) | 1 (reference) | 2318 | 1 (reference) | 1 (reference) |
| Using e-cigarettes | 38 | 0.67 (0.48 to 0.92) | 1.09 (0.79 to 1.51) | 24 | 0.50 (0.34 to 0.75) | 1.01 (0.67 to 1.51) |

**Table S4 Association between smoking status and risk of severe COVID-19 in people without smoking-related disease (N=5,751,061)**

| Category | Number with outcome | Unadjusted HR (95% CI) | HR (95% CI) adjusted for demographic factors and non-smoking related morbidity | HR (95% CI) adjusted for demographic factors, non-smoking-related morbidity, BMI, and smoking-related morbidity |
| --- | --- | --- | --- | --- |
| Outcome hospitalization |  |  |  |  |
| Never smoked | 3218 | 1 (reference) | 1 (reference) | 1 (reference) |
| Stopped smoking | 1241 | 1.23 (1.15 to 1.31) | 1.03 (0.96 to 1.10) | 1.01 (0.94 to 1.08) |
| Smoking 1-9 cigarettes/day | 345 | 0.46 (0.41 to 0.51) | 0.56 (0.50 to 0.63) | 0.58 (0.52 to 0.65) |
| Smoking 10-19 cigarettes/day | 57 | 0.39 (0.30 to 0.51) | 0.49 (0.38 to 0.64) | 0.51 (0.39 to 0.66) |
| Smoking ≥20 cigarettes/day | 32 | 0.51 (0.36 to 0.72) | 0.52 (0.37 to 0.74) | 0.53 (0.37 to 0.75) |
| Outcome ICU admission |  |  |  |  |
| Never smoked | 475 | 1 (reference) | 1 (reference) | 1 (reference) |
| Stopped smoking | 203 | 1.36 (1.16 to 1.61) | 1.13 (0.95 to 1.34) | 1.07 (0.90 to 1.27) |
| Smoking 1-9 cigarettes/day | 36 | 0.33 (0.23 to 0.46) | 0.33 (0.24 to 0.47) | 0.36 (0.26 to 0.51) |
| Smoking 10-19 cigarettes/day | 4 | 0.19 (0.07 to 0.50) | 0.20 (0.08 to 0.54) | 0.22 (0.08 to 0.59) |
| Smoking ≥20 cigarettes/day | 3 | 0.32 (0.10 to 1.01) | 0.29 (0.09 to 0.89) | 0.30 (0.09 to 0.92) |
| Outcome death |  |  |  |  |
| Never smoked | 873 | 1 (reference) | 1 (reference) | 1 (reference) |
| Stopped smoking | 391 | 1.43 (1.27 to 1.61) | 1.03 (0.91 to 1.71) | 1.04 (0.92 to 1.17) |
| Smoking 1-9 cigarettes/day | 76 | 0.37 (0.30 to 0.47) | 0.78 (0.61 to 0.99) | 0.78 (0.61 to 0.99) |
| Smoking 10-19 cigarettes/day | 15 | 0.38 (0.23 to 0.63) | 0.86 (0.52 to 1.44) | 0.86 (0.51 to 1.44) |
| Smoking ≥20 cigarettes/day | 11 | 0.65 (0.36 to 1.17) | 1.00 (0.55 to 1.81) | 1.00 (0.55 to 1.82) |

**Table S5 Association between smoking status and risk of severe COVID-19 in people with smoking-related disease (N=2,118,473)**

| Category | Number with outcome | Unadjusted HR (95% CI) | HR (95% CI) adjusted for demographic factors and non-smoking related morbidity | HR (95% CI) adjusted for demographic factors, non-smoking-related morbidity, BMI, and smoking-related morbidity |
| --- | --- | --- | --- | --- |
| Outcome hospitalization |  |  |  |  |
| Never smoked | 4915 | 1 (reference) | 1 (reference) | 1 (reference) |
| Stopped smoking | 3765 | 1.40 (1.34 to 1.46) | 1.18 (1.13 to 1.24) | 1.08 (1.03 to 1.13) |
| Smoking 1-9 cigarettes/day | 557 | 0.49 (0.45 to 0.54) | 0.71 (0.65 to 0.78) | 0.66 (0.60 to 0.72) |
| Smoking 10-19 cigarettes/day | 63 | 0.32 (0.25 to 0.40) | 0.50 (0.39 to 0.64) | 0.46 (0.36 to 0.60) |
| Smoking ≥20 cigarettes/day | 60 | 0.53 (0.41 to 0.68) | 0.69 (0.54 to 0.89) | 0.63 (0.48 to 0.81) |
| Outcome ICU admission |  |  |  |  |
| Never smoked | 462 | 1 (reference) | 1 (reference) | 1 (reference) |
| Stopped smoking | 315 | 1.25 (1.08 to 1.44) | 1.32 (1.13 to 1.53) | 1.25 (1.07 to 1.46) |
| Smoking 1-9 cigarettes/day | 24 | 0.22 (0.15 to 0.34) | 0.23 (0.15 to 0.35) | 0.25 (0.16 to 0.38) |
| Smoking 10-19 cigarettes/day | 1 | 0.05 (0.01 to 0.38) | 0.06 (0.01 to 0.44) | 0.07 (0.01 to 0.47) |
| Smoking ≥20 cigarettes/day | 4 | 0.38 (0.14 to 1.01) | 0.40 (0.15 to 1.08) | 0.39 (0.14 to 1.04) |
| Outcome death |  |  |  |  |
| Never smoked | 2235 | 1 (reference) | 1 (reference) | 1 (reference) |
| Stopped smoking | 1951 | 1.60 (1.50 to 1.70) | 1.29 (1.21 to 1.38) | 1.19 (1.12 to 1.27) |
| Smoking 1-9 cigarettes/day | 219 | 0.42 (0.37 to 0.49) | 0.87 (0.75 to 1.00) | 0.76 (0.66 to 0.88) |
| Smoking 10-19 cigarettes/day | 25 | 0.28 (0.19 to 0.41) | 0.65 (0.43 to 0.96) | 0.57 (0.38 to 0.85) |
| Smoking ≥20 cigarettes/day | 21 | 0.41 (0.27 to 0.63) | 0.76 (0.49 to 1.17) | 0.65 (0.42 to 1.01) |

***Post hoc sensitivity analysis discussion***

Our sensitivity analysis showed that using smoking status from at least five years previously did not greatly change the strength of association between current smoking and hospitalization and ICU admission, but smoking was associated with increased risk of death from COVID-19. For reverse causation to explain the results would require the illness to be severe enough to substantially increase risk of death but lower the risk of hospitalization and ICU admission from COVID-19, prompt cessation, but remain undiagnosed (and hence not adjusted for) or not be in our adjustment set. This seems an implausible mechanism. Moreover, in the main analysis, 50% of people had their smoking status updated more than 21 months prior to study end, allowing more than 21 months for incipient illness to be investigated and diagnosed, which ought to be enough to exclude reverse causation. The sensitivity analysis allowed an average of six years to do so. Moreover, if reverse causation explained the results in our main analysis, reverse causation ought to have depressed all-cause mortality, which was elevated in people who smoked.

Adjusting for age as a non-linear term did not meaningfully influence the association between smoking and severe COVID-19. Stratifying by the presence of or absence of smoking-related disease did not change the association, with each group producing estimates similar to those of the combined group.

**E values for all exposure-outcome associations and E-values from a recent epidemiological study of the consequences of smoking**

**Table S6 E-values corresponding to Table 2**

|  | HR (95% CI) adjusted for demographic factors, non-smoking-related morbidity, BMI, and smoking-related morbidity | E-values for point estimate and 95% confidence intervals |
| --- | --- | --- |
| Outcome hospitalization |  |  |
| Never smoked | 1 (reference) |  |
| Stopped smoking | 1.07 (1.03 to 1.11) | 1.34 (1.21 to 1.46) |
| Smoking 1-9 cigarettes/day | 0.64 (0.60 to 0.69) | 2.50 (2.72 to 2.26) |
| Smoking 10-19 cigarettes/day | 0.49 (0.41 to 0.59) | 3.50 (4.31 to 2.78) |
| Smoking ≥20 cigarettes/day | 0.61 (0.49 to 0.75) | 2.66 (3.50 to 2.00) |
| Outcome ICU admission |  |  |
| Never smoked | 1 (reference) |  |
| Stopped smoking | 1.17 (1.04 to 1.31) | 1.62 (1.24 to 1.95) |
| Smoking 1-9 cigarettes/day | 0.31 (0.24 to 0.41) | 5.91 (7.80 to 4.31) |
| Smoking 10-19 cigarettes/day | 0.15 (0.06 to 0.37) | 12.81 (32.83 to 4.85) |
| Smoking ≥20 cigarettes/day | 0.36 (0.17 to 0.76) | 5.00 (11.24 to 1.96) |
| Outcome death |  |  |
| Never smoked | 1 (reference) |  |
| Stopped smoking | 1.17 (1.10 to 1.24) | 1.62 (1.43 to 1.79) |
| Smoking 1-9 cigarettes/day | 0.79 (0.70 to 0.89) | 1.85 (2.21 to 1.50) |
| Smoking 10-19 cigarettes/day | 0.66 (0.48 to 0.90) | 2.40 (3.59 to 1.46) |
| Smoking ≥20 cigarettes/day | 0.77 (0.54 to 1.10) | 1.92 (3.11 to 1.43) |

The paper is Pirie K, Peto R, Reeves G, Green J, Beral V. The 21^st^ century hazards of smoking and the benefits of stopping: a prospective study of one million women in the UK

<https://www.thelancet.com/journals/lancet/article/PIIS0140-6736(12)61720-6/fulltext#secd5663140e303>

**Table S7 Established smoking-related disease and their E-values from the paper**

| Smoking-related disease | E-values of point estimate and 95% confidence intervals |
| --- | --- |
| Cancer of the pancreas | 3.97 (3.52 to 4.48) |
| Cancer of the kidney | 3.64 (2.90 to 4.50) |
| Cancer of the stomach | 3.31 (2.60 to 4.17) |
| Pulmonary fibrosis | 2.43 (1.57 to 3.50) |
| Leukaemia | 1.97 (1.37 to 2.62) |
